# Targeting PI3K Signaling in Acute Lymphoblastic Leukemia

**DOI:** 10.3390/ijms20020412

**Published:** 2019-01-18

**Authors:** Vanessa Edna Sanchez, Cydney Nichols, Hye Na Kim, Eun Ji Gang, Yong-Mi Kim

**Affiliations:** 1 Department of Pathology, Keck School of Medicine of University of Southern California, Los Angeles, CA 90033, USA; vanesses@usc.edu; 2 Department of Pediatrics, Division of Hematology, Oncology, Blood and Marrow Transplantation, Children’s Hospital Los Angeles, University of Southern California, Los Angeles, CA 90027, USA; hyekim@chla.usc.edu (H.N.K.); ejiang@chla.usc.edu (E.J.G.); 3 New York Medical College School of Medicine, Valhalla, NY 10595, USA; cnichols5@student.nymc.edu

**Keywords:** acute lymphoblastic leukemia (ALL), cell adhesion mediated drug resistance (CAM-DR), PI3K/AKT, PI3Kδ

## Abstract

Adhesion of acute lymphoblastic leukemia (ALL) cells to bone marrow stroma cells triggers intracellular signals regulating cell-adhesion-mediated drug resistance (CAM-DR). Stromal cell protection of ALL cells has been shown to require active AKT. In chronic lymphocytic leukemia (CLL), adhesion-mediated activation of the PI3K/AKT pathway is reported. A novel FDA-approved PI3Kδ inhibitor, CAL-101/idelalisib, leads to downregulation of p-AKT and increased apoptosis of CLL cells. Recently, two additional PI3K inhibitors have received FDA approval. As the PI3K/AKT pathway is also implicated in adhesion-mediated survival of ALL cells, PI3K inhibitors have been evaluated preclinically in ALL. However, PI3K inhibition has yet to be approved for clinical use in ALL. Here, we review the role of PI3K in normal hematopoietic cells, and in ALL. We focus on summarizing targeting strategies of PI3K in ALL.

## 1. Introduction

Chemotherapeutic resistance in adults with acute lymphoblastic leukemia (ALL) remains a major problem [1]. Although survival rates have improved in childhood ALL, the problem of relapse of ALL after chemotherapy remains unsolved [2]. The bone marrow environment has been shown to promote cell adhesion-mediated drug resistance (CAM-DR) in leukemia cells, which may contribute to relapse. The bone marrow (BM) is the most frequent site of relapse in ALL [3,4], and BM relapse is associated with a worse prognosis than isolated extramedullary relapse [3,5]. These findings point to a protective role of the BM as a safe haven for drug-resistant ALL cells. The BM is comprised of a variety of different types of cells and molecules including fibroblastic stromal cells, endothelial cells, growth factors, cytokines, and hormones. In the event of malignant cell transformation, biological changes within the BM occur; this is seen in the stromal cells, which lead to activation of pro-survival signals of leukemia cells. In vitro studies show that contact between leukemia and stromal cells promotes cell adhesion-mediated drug resistance (CAM-DR) [6,7], which prevents apoptosis of ALL cells [8,9,10]. Therefore, identifying the underlying mechanism of this survival promoting CAM-DR is critical to overcoming chemotherapeutic drug resistance.

**Figure 1:**
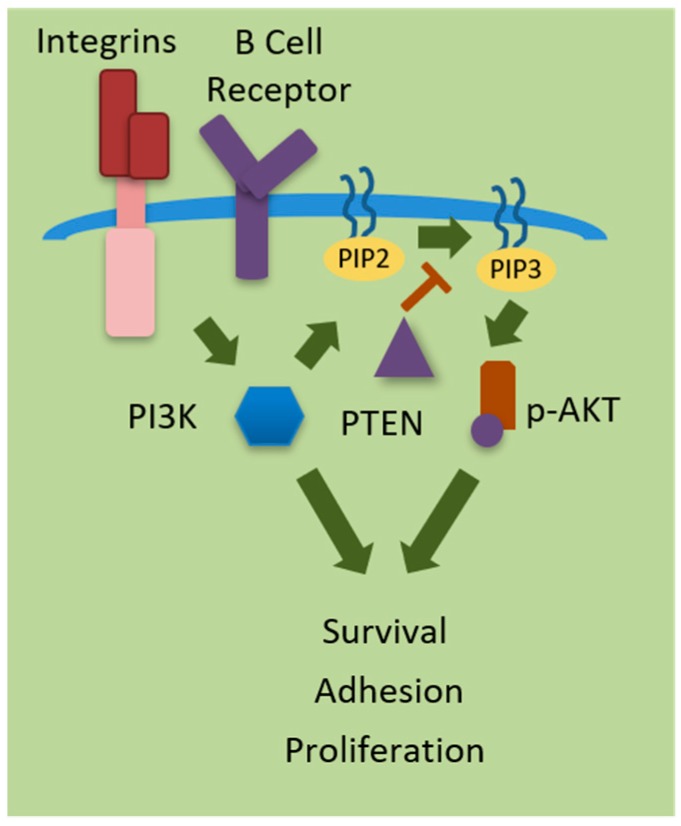
Phosphoinositide 3-kinases (PI3Ks) regulate cellular processes including cell survival, adhesion, and proliferation. PI3K is activated by receptor tyrosine kinases (RTKs), Integrins, or G-protein coupled receptors (GPCRS) at the surface of the cell. PI3K phosphorylates phosphatidylinositol-diphosphate (PIP2) into phosphatidylinositol triphosphate (PIP3), which in turn activate the serine/threonine kinase AKT regulations survival, adhesion and proliferation. PTEN (phosphatase and tensin homolog deleted from chromosome 10) negatively regulates PI3K signaling. 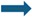 = Activation; ⊥ = Inhibition.

Integrin-linked kinase (ILK) has been implicated in acute myeloid leukemia cells as a key player upstream of AKT in the PI3K prosurvival pathway induced by bone marrow-derived stromal cells [11] (see Figure 1). Activation of the phosphatidylinositol-3-kinase (PI3K) pathway including AKT is associated with poor prognosis and drug resistance in pediatric pre-B ALL, as well as decreased chemotherapy-driven apoptosis in vitro [12]. PI3K pathway activation by cell surface receptors is directly mediated by class I isoforms p110α, p110β, p110δ, and p110γ. Inhibition of the PI3K/AKT pathway leads to decreased cell proliferation in chronic lymphoblastic leukemia (CLL) [13] and acute myeloid leukemia (AML) [14,15]. Recently, idelalisib has been found to be active against pre-B acute lymphoblastic leukemia (pre-B ALL) [16]. Since PI3Kδ has been linked to hyperactivation of the PI3K pathway leading to uncontrollable cell proliferation, disrupting this type of cell mediated signaling has become an attractive target in many diseases, including ALL. An array of targeted therapies is in continual development as well as in clinical trials. PI3K inhibitors may restore sensitivity to other treatments when administered as part of combination regimens [17]. Targeting certain receptors or subunits of PI3K have proven to be beneficial. However, side effects for some inhibitors have been described [18,19]. In this review we will overview the significance of the Phosphatidylinositol 3- kinase (PI3K) pathway in normal B cells and ALL, as well as preclinical and clinical evaluations of therapeutic drugs targeting PI3K in the context of ALL.

## 2. Isoforms of PI3K

The PI3K family consists of a group of enzymes known as a key transducer of signals which control the proliferation, differentiation, self-renewal, and survival of hematopoietic stem cells (HSCs). There are three separate classes of PI3Ks, categorized depending on their composition of subunits and functional role in phosphorylating inositol. The three PI3K classes phosphorylate the 3′-position hydroxyl of the D-myo-inositol head group to generate different forms of phosphoinositide. Of the three, only Class I can produce PIP3. All PI3Ks have a motif composed of a C2 domain (likely for membrane binding), a helical domain, and a catalytic kinase domain. The presence of additional protein domains aids in the differentiation of PI3K classes.

Class I is most frequently correlated with the development of cancer. Class I PI3Ks contain catalytic subunits that are categorized into four subunits: p110α, p110β, p110δ (class1A), and p110γ (class1B). Each of the p110 isoforms share some overlap while maintaining distinct functions. They are tissue specific and are therefore being studied for the development of localized drug targets for the treatment of hematopoietic malignancies. The p110α and p110β isoforms of Class I PI3K molecules are universally expressed in all tissues [20]. Furthermore, breast and cervical cancers have been associated with the p110α catalytic subunit [20]. Overexpression of the *PIK3CA* gene encoding the p110α catalytic subunit is also seen in primary AML and multiple myeloma patient samples. PI3K p110δ is encoded by *PIK3CD* gene and is enriched in leukocytes [21,22]. P110δ and p110γ have been shown to play major roles in hematological malignancies. The p110γ subunit is involved in the cell motility of macrophages, and studies inhibiting this subunit have shown a reduction in the proliferation of lung cancer cells in pulmonary fibrosis [23]. It is important to note that none of the isoforms are exclusively expressed in leukocytes.

Class II PI3Ks are monomers categorized into 3 categories, PI3KC2α, PI3KC2β, and PI3KC2γ. There are no known regulatory subunits, although class II enzymes have been shown to interact with possible adaptor proteins. The catalytic portion produces phosphatidylinositol-3-phosphate and phosphatidylinositol-3,4-biphosphate. These proteins are activated by growth hormones, chemokines, and a variety of stimulants at the cell surface [22]. PI3KC2α and PI3KC2β are ubiquitously expressed throughout the body, while PI3KC2γ is seen in the liver, prostate, and breast [24].

Class III PI3K is a heterodimer consisting of a catalytic, Vps34, and a regulatory, Vps15, subunit. This type of PI3K produces phosphatidylinositol-3-phospate and is also expressed ubiquitously [20]. It plays a role in trafficking molecules to vesicles for protein sorting, maturation, autophagosome formation, autophagy flux, and cytokinesis [20,25].

## 3. Regulation of PI3K Signaling

Phosphatidylinositol 3- kinase (PI3K) is activated by receptor tyrosine kinases (RTKs) or G-protein coupled receptors (GPCRs) at the surface of the cell. PI3K phosphorylates phosphatidylinositol-diphosphate (PIP2) into phosphatidylinositol triphosphate (PIP3). PIP3 is a second messenger and serves as a docking site for proteins with pleckstrin-homology (PH) domains, including phosphoinositide-dependent kinase 1 (PDK1) and its downstream target, protein kinase B (AKT). When AKT binds and is activated, pro survival signaling cascades are initiated, supporting the reduction of apoptosis while increasing cell motility, survival, and growth [22]. Regulation of the PI3K pathway is largely due to the negative regulator phosphatase and tensin homolog (PTEN), a lipid phosphatase. PTEN dephosphorylates PIP3, thereby preventing AKT activation, essentially turning off the PI3K pathway. The inactivation of PTEN has been shown to be highly prevalent in several cancers including T-cell acute lymphoblastic leukemia (T-ALL) [26]. In fact, The PI3K pathway is activated in 92% of T-ALL cell lines and in 81% of primary T-ALL samples, as reported by Yuan et al. [27]. PTEN loss of function due to gene mutations or deletions is seen in over 20% of T-ALL patients. Ultimately, this leads to the hyperactivation of PI3K and its downstream effectors, thereby enhancing a pro-tumoral environment for cancerous cells, aiding in proliferation, progression, and survival [20].

PI3K pathway activation can also be achieved through the receptor tyrosine kinases (RTKs) such as platelet derived growth factor receptor (PDGFR), insulin-like growth factor- I receptor (IGF-IR), and Fms-Related Tyrosine Kinase 3 (FLT3). There are consistent activations of the PI3K pathway due to mutations, duplications, and overexpression of ligands, all of which are associated with poorer prognosis [22]. GPCRs specific to p110δ and p110γ subunits activate the PI3K pathway as well. Micro-environmental factors may play a role as well, promoting a positive feedback loop mechanism for the continual activation of the PI3K pathway. IL-6 and stromal derived factor-1 (SDF-1) have been shown to be significant in other hematological cancers by stimulating the tumor cell and bone marrow microenvironment interactions that promote cell survival [22].

## 4. Role of PI3Kδ in Normal B-Cells

B lymphocytes develop through several stages originating in the bone marrow. Immature B cells travel from the bone marrow to the spleen, where they complete maturation, becoming follicular or marginal zone B cells. Naïve B cells (B cells that have not yet been exposed to antigen) express B cell antigen receptor (BCR), a transmembrane receptor composed of immunoglobulin (Ig) molecules; 2 Ig heavy (IgH) chains (μ or δ isotype) and 2 Ig light chains (κ or λ isotype) [28]. This receptor contains an immune receptor tyrosine-based activation motif (ITAM) within its cytoplasmic domain. PI3K is a central nodal molecule in facilitating these signals in leukocytes as it is downstream of BCR. In B cells PI3Kδ plays a role downstream of both BCR and integrins, each of which are involved in interactions between leukemia cells and their surroundings.

BCR can be activated via antigenic stimulation, which initiates a complex network of signaling cascades leading to cell survival, proliferation, or differentiation depending on the type of antigen, involvement of co-stimulatory molecules, and maturation status of the cell [29]. Integrin interactions mediate cell-to-cell interactions and play a tremendous role in hematopoietic cell development as this process relies on immature lymphocyte contact with bone marrow. Similarly, integrins (for example, integrin alpha 4) mediate the necessary contact between bone marrow and leukemia cells for the survival and proliferation of this cancer in patients [30]. PI3Kδ is an important conduit for information from these cell surface molecules to many established pathways including NF-κB, JAK/STAT, and AKT/mTOR routes for signaling.

More specifically, binding of antigen to BCR initiates phosphorylation of tyrosine residues located within the cytoplasmic domain. This phosphorylation activates PI3K, now able to propagate the signal by phosphorylating the 3′-hydroxyl group of phosphatidylinositol-diphosphate (PIP2), thereby converting it to the second messenger, phosphatidylinositol-triphosphate (PIP3) [26]. Acting as a docking site for proteins with pleckstrin-homology (PH) domains, PIP3 advances the signal by activating phosphoinositide-dependent kinase 1(Pdk1), which then phosphorylates the downstream effector protein kinase B (AKT) on Thr308 [28]. Further amplification of the AKT signaling pathway affects proteins such as Bax, preventing apoptosis; mTOR, causing increased survival, proliferation, and migration; and FOXO, a negative PI3K regulator tumor suppressor, which is destroyed by proteasomes [22]. Along with cell-cell contact, cytokines from T cells or immune cells and the binding of antigen to BCR promotes B cell proliferation and differentiation into long-lived antibody-secreting plasma cells and memory B cells [31,32].

Naïve mature B cell numbers are regulated by several receptors such as BCR [33]. Studies have shown key survival signals from BCR are transduced by PI3K [31]. Ablation of BCR in mature B cells in vivo resulted in cells undergoing apoptosis. Activation of the PI3K signaling pathway rescued B cells. PI3K, therefore, is a critical component in the determination of B cell development [31]. At the mature state, the predominant player in B-cells is the PI3Kδ isoform [20]. Since B-cells exhibit high levels of PI3Kδ in comparison to the three other isoforms, it is the preferred isoform engaged with p85α as the heterodimer activated by BCR [28]. Therefore, PI3Kδ acts in an agonistic manner and is essential for the survival of mature B cells. BCR ablation studies not only lead to B cell death, but showcase the ability for rescue by the deletion of negative regulators of the PI3K pathway, PTEN or FOXO [31].

As previously stated, the p110δ isoform is predominantly detected in leukocytes [34] and furthermore plays a role in B cell growth and function. Deleted or mutated PI3Kδ mice exhibit a B-cell defect with decreased mature B-cell numbers and impaired antibody production. PI3Kδ knockout mice also show less AKT phosphorylation (p-AKT) in activated B-cells [35]. It has been demonstrated that p110α and p110δ cooperate in suppressing class switch recombination (CSR) and that PTEN overexpression stimulates CSR in mature B cells [36]. It mediates signaling between RTKs and the tyrosine-based activation motif (ITAM)-containing proteins. PI3Kδ has shown to be necessary for the proper function of mast cells, neutrophils, T cells, and B cells, as its inhibition reveals PI3K dependence [24]. PI3Kδ is also crucial for normal B cell motility [37]. The PI3K signaling pathway has been implicated in in B cell activation, differentiation, and survival [38], as well as in regulating cell metabolism [39].

The significance of PI3Kδ signaling in human hematopoiesis is further emphasized by the detrimental clinical features of Activated PI3Kδ Syndrome (APDS), an autosomal dominant immune disorder. Multiple mutations have been identified in the *PIK3CD* gene encoding the p110δ catalytic domain (APDS type 1) and in the *PIK3R1* gene encoding the p85α regulatory domain which lead to activation of PI3Kδ [40]. Impaired lymphocyte differentiation (normally mediated by PI3K signaling) renders patients with this condition especially susceptible to chronic bacterial respiratory tract infections and viral infections like Epstein–Barr Virus and Cytomegalovirus. Additionally, APDS patients experience autoimmune and inflammatory disorders as well as higher incidences of lymphomas linked to PI3K signaling dysregulation [41].

Interestingly, in addition to conventional antibiotic prophylaxis and immunosuppressive therapy, APDS patients have shown to benefit from PI3Kδ inhibitors to address the overactivation of this signaling molecule. Two clinical trials are currently evaluating the use of the PI3Kδ inhibitors leniolisib (NCT02435173) and nemiralisib (NCT02593539) in APDS patients. Leniolisib has yielded normalization of circulating lymphocytes, serum IgM levels, and inflammatory markers as well as reduction in lymphadenopathy and splenomegaly due to abnormal lymphoproliferation [42].

The recent linkage of the etiology of APDS with mutations in PI3Kδ solidifies our understanding of the essential role of PI3Kδ signaling in proper immune cell function. Furthermore, the use of PI3Kδ inhibitors in this disorder supports potential far reaching applications of these drugs in immune deficiencies even outside the context of cancer.

## 5. Role of PI3Kδ in ALL

In general, PI3K activated by receptor tyrosine kinases or G protein coupled receptors at the cell surface phosphorylates PIP2 to PIP3. Activation of the regulatory subunit of PI3K, p85, removes inhibition of the catalytic domain, p110. PIP3 leaves the plasma membrane to phosphorylate AKT or other signaling molecules, causing stimulation of various intracellular pathways and ultimately leading to cell survival, proliferation, differentiation, or other physiological changes. In the context of cancer, the PI3K pathway frequently undergoes stimulatory alterations including activating mutations in upstream RTKs, the downstream molecules AKT, and in PI3K itself, as well as loss of function mutations in the negative regulator (PTEN) [43].

Specifically in ALL, mutations have been observed in exon 9 and 20 of *PIK3CA* (encoding the p110α catalytic subunit), exon 12 and 13 of *PIKRA* (encoding the p85α regulatory subunit), and exon 2 of AKT1 [44]. These mutations are more commonly observed in T-ALL relative to B-ALL subtypes [45]. Intriguingly, PTEN is not usually mutated in B-ALL in contrast to T-ALL, although increased levels are observed. However, elevated PTEN does not abrogate PI3K signaling as would be expected due to concurrent increases in CK2, a kinase that phosphorylates and inactivates these PTEN molecules [46].

PI3Kδ signaling is central to cell–cell interactions involving ALL. Adhesion of acute lymphoblastic leukemia (ALL) cells to bone marrow stroma cells triggers intracellular signals regulating cell-adhesion-mediated drug resistance (CAM-DR) [30,47]. Stromal cell protection of ALL cells has been shown to require active AKT [48]. Tabe et al. showed that ILK/AKT is a signaling pathway critical for the survival of leukemic cells [11]. This group demonstrated the activation of several components necessary for the survival of leukemic cells such as ILK/AKT, extracellular signal-regulated kinase 1/2 (ERK1/2), signal transducers and activators of transcription 3 (STAT3), as well as Notch1/Hes in a coculture system of leukemic NB4 cells with bone marrow-derived stromal mesenchymal stem cells (MSCs). Blockade of PI3K or ILK signaling with pharmacologic inhibitors LY294002 or QLT0267 resulted in the induction of apoptosis in both leukemic cell lines and in primary acute myeloid leukemia samples. Muranyi et al. showed that targeting integrin linked kinase (ILK) and FMS-like tyrosine kinase-3 (FLT3) with an inhibitor of ILK and FLT3, OLT0267, is cytotoxic to acute myeloid leukemia stem cells using a long-term suspension culture system and a NOD/SCID mouse leukemia-initiating assay [49].

PI3Kδ has been linked to hyperactivation of the PI3K pathway, leading to uncontrollable cell proliferation. Thus, disrupting this type of cell mediated signaling has become an attractive target in many diseases, including ALL [18,50]. Activation of the PI3K/AKT pathway is associated with poor prognosis and drug resistance in pediatric pre-B ALL [12]. Therefore, an array of targeted therapies established in clinical practice and various other preclinical inhibitors are under continual investigation and development. Inhibition of the PI3K/AKT pathway has led to decreased cell proliferation of several hematological malignancies including CLL [13,51,52,53,54] and AML [14,15,55]. In CLL, PI3Kγ inhibition reduced CLL adhesion to stromal cells to a similar extent as the PI3Kδ inhibitor idelalisib [56].

Biological and clinical distinction of ALL subtypes can be challenging to identify partly because of a variety of genetic aberrations. Specific genetic alterations can be used as hallmarks or targets for the characterization of subtypes of ALL, yet are difficult to detect using standard diagnostic methods [57]. Advanced genetic testing is, therefore, beneficial to enhance classification of ALL subtypes through the identification of specific genetic alterations. In a previous study, the characterization of the genetic landscape through genomic profiling and transcriptome sequencing was utilized to conclude the frequency of genetic alterations due to genetic fusions. Gene fusions involving tyrosine kinase genes, such as v-abl-Abelson murine leukemia viral oncogene homologue 1 (*ABL1*), v-abl- Abelson murine leukemia viral oncogene homologue 2 (*ABL2*), Colony Stimulating Factor 1 Receptor (*CSF1R*), and Platelet Derived Growth Factor Receptor Beta (*PDGFRB*), were shown to induce cytokine-independent proliferation as well as activate phosphorylated Signal Transducers and Activators of Transcription (STATs) [58]. The transcription factor, STAT5, has been shown to be involved in cross talk with PI3K/AKT pathway, providing sufficient signaling for cell survival, proliferation, and overriding cell death programs [59].

Gene fusion aberrations were highly associated with Ph-like ALL patients and all were shown to activate STAT5 [58]. Activation of STAT5 regulates the transcription of p85 and p110 subunits of PI3K and therefore serves as a driver of oncogenesis in Ph-like ALL patients [58,60,61]. This study demonstrated that the *ABL1, ABL2*, and *CSFR1R* fusions were sensitive to ABL class inhibitors, like imatinib and dasatinib, current FDA-approved tyrosine kinase inhibitors [58]. *BCR-ABL1* gene fusion subtypes were demonstrated to also be sensitive and have promising sensitivity to the use of tyrosine kinase inhibitors [57]. Thus, current FDA approved PI3K inhibitors can be used in conjunction with current therapy to improve the outcome of ALL, as seen in Ph-like ALL. Subtyping of ALL via advance genetic profiling methods can better identify aberrations sensitive to current FDA-approved PI3K inhibitors in order to improve therapeutic approaches in the treatment of ALL.

## 6. Preclinical Inhibition of PI3K/PI3Kδ in ALL

**Figure 2:**
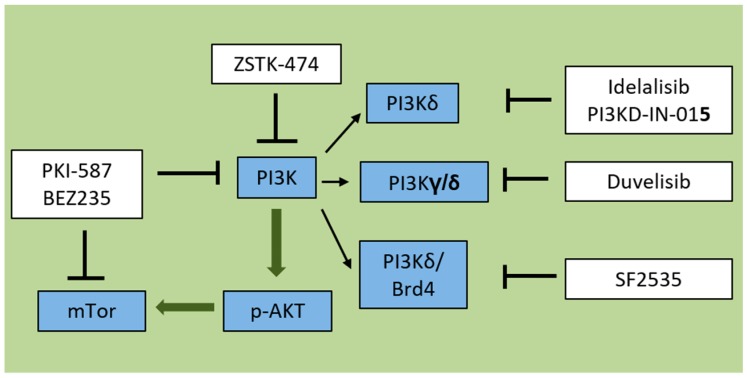
PI3K pathway inhibitors target one or more aspects of PI3K signaling. PKI-587 and BEZ235 are dual PI3K/mTOR inhibitors. ZSTK-474 is a pan-PI3K inhibitor. Idelalisib and PI3K-IN-015 are each targeting PI3Kdelta isoforms. Duvelisib is a dual PI3K gamma/delta inhibitor. SF2535 is a novel PI3Kdelta/BRD4 inhibitor. → = Include 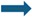 = Activation ⊥ = Inhibition.

There is considerable interest in preclinical and clinical investigation of PI3K inhibitors including pan-PI3K inhibitors targeting all four isoforms of class I PI3K, as well as isoform-selective inhibitors (Figure 2). Novel PI3K inhibitors are under further evaluation for lymphoma and other cancer types, which are not discussed here [18]. Here, we review PI3K inhibitors in preclinical evaluation for the treatment of ALL (Table 1). Some of these are in clinical development, which is summarized in Table 2 and Table 3.

Idelalisib (also known as CAL-101 or GS-1101) has been found to be active against pre-B acute lymphoblastic leukemia (pre-B ALL) [16,52,62,63,64,65]. PI3Kδ inhibition using idelalisib decreases p-AKT levels and prevents migration towards SDF-1α [16]. Kruth et al. have shown that idelalisib interrupts a double-negative feedback loop, enhancing glucocorticoid regulated transcription to synergistically kill even highly resistant B-ALL with diverse genetic profiles. Supportive of this finding are the results of Eldfors et al. indicating that idelalisib is effective against TCF3-PBX1 B-Cell Precursor Acute Lymphoblastic Leukemia (BCP-ALL). This group also demonstrated that TCF3-PBX1 controls expression of *PIK3CD*, the gene encoding p110δ [66]. Tasian et al. showed that PI3K/mTOR inhibition potently decreases ALL cell burden in vivo and thus further enhances antileukemia activity [63]. It has also been demonstrated that CAL-101 is toxic against NALM6 pre-B-ALL cells and that the observed growth-suppressive effect is mediated, at least partially, by G1 arrest as a result of upregulated p21 [67]. Idelalisib reduces transit of ALL cell lines along bridging vessels, blast counts in the cerebrospinal fluid, and CNS disease in a murine ALL xenograft [68]. As PTEN, PI3K/AKT and Notch pathways are frequently altered in T-ALL, Yuan et al. recently demonstrated that ShRNA for *PIK3CD* and CAL-101 reduces growth and increases apoptosis of T-ALL cells [27].

Copanlisib is a pan-P13K inhibitor characterized by enhanced affinity due to its planar conformation, allowing for deeper penetration into the p110 subunit binding pocket. This increased affinity has yielded Copanlisib higher specificity against CLL and Diffuse Large B-Cell Lymphoma (DLBCL) cell lines when used in nanomolar concentrations [69].

PI3KD-IN-015, a novel PI3Kδ inhibitor, is purported to moderately impact the proliferation of a variety of B-cell related cancer cell lines through down-regulating PI3K signaling leading to apoptosis and autophagy. More specifically, authors demonstrated that PI3K-IN-015 inhibits phosphorylation of AKT at position T308, usually carried out by PI3K. This newer PI3Kδ inhibitor exhibits an IC50 of 13nM compared with 2.3 nM by CAL-101, a more well-established PI3Kδ isoform inhibitor [70].

Duvelisib is also known as ABBV-954, INK-1197, or IPI-145. It is a dual p110γ/p110δ inhibitor which has been shown to decrease viability and induce apoptosis and autophagy in BCR-ABL positive B-ALL when combined with imatinib or nilotinib [71]. In this study, Ultimo et al. also showed that the pan-PI3K inhibitor ZSTK474, p110α inhibitor BYL719, and dual p110 γ/δ inhibitor IPI145, in combination with tyrosine kinase inhibitors, induced apoptosis in BCR-ABL positive B-ALL cell lines. Interestingly, in another study, this dual γ/δ PI3K p110 inhibitor rendered B-ALL cells more susceptible to Dexamethasone [72]. Furthermore, experiments in a murine xenograft model using primary CLL samples showed impaired homing of leukemic B and T cells from peripheral blood to splenic compartments, indicating a role for Duvelisib in impairing signaling between micro-environmental and tumor cells [73].

SF2535 is a dual PI3Kδ/BRD4 inhibitor which ultimately downregulates MYC levels. BRD4 is a member of the BET family of proteins which bind acetylated lysines at promoter and enhancer regions of a variety of genes including the transcription factor MYC; thus, it blocks MYC expression and activation. Additionally, PI3K activation usually prevents MYC degradation via of glycogen synthase kinase 3 β (GSK3 β), therefore, inhibition of PI3K by SF2535 also increases MYC degradation [74]. It has recently been evaluated in primary pre-B ALL and has shown efficacy in inducing apoptosis in vitro [75].

PKI-587 is also known as Gedatolisib or PF 05212384. It is a dual PI3K/mTOR pathway inhibitor, which inhibits proliferation in T-ALL cell lines in vitro and enhances survival in immune-deficient mice engrafted with the T-ALL cell line CCRF-CEM in vivo. More specifically, it delayed tumor progression without inducing significant weight loss, suggesting, at least in mice, a favorable toxicity profile supporting further investigation [76].

BEZ235, also named Dactilosib, is a PI3K/mTOR small molecule inhibitor which led to synergistic anti-proliferative effects on both Philadelphia chromosome positive (Ph+) and negative ALL cells when combined with the pan-BCL-2 blocker obatoclax. The use of primary patient samples spanning both Ph+ and Ph− status addresses the important question of the applicability of these drugs to patients with variable cytogenetics [77].

ZSTK-474, a PI3K pan-inhibitor, decreased T-ALL cell survival and induced apoptosis in nelarabine-resistant T-ALL cells. Investigators showed dephosphorylated AKT and ERK1/2 in response to the combination of these two drugs. They also demonstrate increased Bax/Bak expression, suggesting a role for this combination in directly impacting the apoptosis pathway in tumor cells [78]. ZSTK-474 showed the highest cytotoxic effects when compared to both selective isoform inhibition (using the p110α inhibitor A-66, p110β inhibitor TGX-221, p110δ inhibitor CAL-101, and p110γ inhibitor AS-605240) and the dual p110γ/δ inhibition (using IPI-145) in T-ALL cell lines [79]. When examining the mechanism of action of ZSTK-474, investigators observed a decrease in phosphorylation of AKT at both possible sites (Thr308 and Ser473). 

## 7. PI3K Targeting Drugs in Clinical Trials

Although there are no FDA-approved PI3K inhibitors for acute leukemias, so far, three PI3K inhibitors have gained approval for treatment of other cancers: Idelalisib, Copansilib, and Duvelisib (Table 2).

Idelalisib (Zydelig), was approved for patients with Indolent Non-Hodgkin Lymphoma (iNHL) in July 2014 [80]. This approval was based on findings from a single-arm phase II study, in which single-agent idelalisib showed an overall response rate (ORR) of 54% for patients with follicular lymphoma and 58% for those with SLL [55,81]. Recently, clinical use of idelalisib has been associated with pulmonary adverse events [82,83] and other side effects, and thus requires close monitoring [84,85].

Copanlisib was FDA approved in September 2017 as a second PI3K inhibitor. Its higher affinity for the p110 subunit of PI3K has allowed higher specificity and decreased gastrointestinal toxicity compared with idelalisib [69] (Aliqopa; Bayer). It is a pan-class I PI3K inhibitor with predominant activity against PI3Kα and PI3Kδ isoforms and has been recently approved for patients with relapsed follicular lymphoma [86,87]. Clinical trials are currently underway examining copanlisib’s potential application in multiple subtypes of non-Hodgkin lymphoma [69].

Duvelisib is an oral dual inhibitor of PI3Kδ and PI3Kγ for hematologic malignancy treatment [88,89,90]. The FDA approved duvelisib (Copiktra; Verastem) in September 2018 for patients with relapsed/refractory chronic lymphocytic leukemia/small lymphocytic lymphoma (CLL/SLL) who have received at least two prior therapies. The agency also granted the drug accelerated approval for patients with relapsed/refractory follicular lymphoma (FL) following at least two other therapies [91,92]. The FDA approval was based on favorable outcomes of the phase III DUO and the phase II DYNAMO trials. In DUO, patients with CLL/SLL treated with the drug had a median PFS of 16.4 months and an ORR of 78%, compared with 9.1 months and 39% in patients who received the CD20 monoclonal antibody ofatumumab (Arzerra; Novartis) [89]. In DYNAMO, patients with FL treated with the drug had an ORR of 41% with a good safety profile as the majority of adverse events were low grade (≤Grade 2) [93].

## 8. PI3K Inhibitors in Clinical Trial in ALL

As efforts to develop new pre-clinical and clinical inhibitors of the PI3K pathway are ongoing, few PI3K targeting drugs are under clinical evaluation for the treatment of acute lymphoblastic leukemia (Table 3). Buparlisib (BKM120) is a pan-class I PI3K inhibitor that showed modest efficacy and was tolerable in advanced acute leukemias (12 acute myeloid leukemia, 1 acute lymphoblastic leukemia, and 1 mixed phenotype leukemia) [94]. Severe adverse events were reported for buparlisib in combination with fulvestrant as tested in breast cancer patients [95].

BEZ235 (also known as dactolisib) is under clinical evaluation at the Johann Wolfgang Goethe University Hospital in Germany for use in ALL and other hematological malignancies (NCT01756118). 

## 9. Discussion

Resistance to multimodal chemotherapy continues to limit the prognosis of ALL, which occurs in part through a process called cell adhesion-mediated drug resistance that hinges on ALL cell adhesion to the stroma. This interaction elicits pro-survival signaling that allows leukemia cells to withstand therapy. A key signaling cascade in this process is PI3K/AKT pathway. In the past few years, three PI3K inhibitors have been approved by the FDA for hematological malignancies. While the preclinical data of PI3K inhibitors in B-ALL look promising, as of today they are not in clinical use in B-ALL patients. Clinical use of PI3K inhibitors is associated with reported toxicities for many drugs [19]. The underlying mechanism of these PI3K inhibition-associated side effects is not well understood, but likely related to the widespread role for PI3K in intracellular signal transduction. Especially in the mostly pediatric patient population affected by ALL, side effects of treatment can be faced well into adulthood for many children, highlighting the importance of highly specific drugs with minimal toxicity.

B-ALL patients may benefit from the application of current PI3K inhibitors to this disease process, or the development of novel PI3K inhibitors. The persistent question is how to determine the best target in the PI3K pathway since none of the isoforms is exclusively expressed in leukocytes. While we have summarized some molecular alterations associated with the PI3K pathway, it remains to be determined if certain PI3K targeted therapies benefit all or some patient populations. Few applications of targeted therapies in ALL have been reported so far (Table 3). 

Observed toxicities in several clinical trials targeting PI3K warrant further investigations to understand these side effects in order to address or prevent them. Further studies are needed to evaluate PI3K inhibitors in vitro and in vivo to identify safe and efficient clinical candidates and to determine specific targeted therapy for ALL patients.

## Figures and Tables

**Table 1:** PI3K inhibitors preclinically evaluated in acute lymphoblastic leukemia (ALL).

Therapeutic Drug	Target (s)	Preclinical Model	Reference
Idelalisib (CAL101)	PI3Kδ inhibitor	B-ALL	[16,52,62,63,64,65]
PI3KD-IN-015	PI3Kδ inhibitor	B-ALL	[70]
Duvelisib (ABBV-954, INK-1197, IPI-145)	PI3Kδ/PI3Kγ inhibitor	B-ALL	[72]
SF2535	PI3Kδ/BRD4 inhibitor	B-ALL	[75]
PKI-587	PI3K/mTOR inhibitor	T-ALL	[76]
Dactolisib (BEZ235)	PI3K/mTOR inhibitor	B-ALL	[77]
ZSTK-474	PI3K pan- inhibitor	T-ALL	[78,79]

**Table 2:** FDA-approved PI3K inhibitors.

Therapeutic Drug	Target (s)	Clinical Trial Status	Developer
Idelalisib (CAL101)	PI3Kδ inhibitor	FDA Approved	Gilead Sciences
Copanlisib	Pan-class I PI3K inhibitor	FDA Approved	Aliqopa; Bayer
Duvelisib (ABBV-954, INK-1197, IPI-145)	PI3Kδ/PI3Kγ inhibitor	FDA Approved	Copiktra; Verastem

**Table 3:** PI3K inhibitors in clinical trial in ALL.

Therapeutic Drug	Target (s)	Clinical Trial Status	Developer	Clinical Trial Number
Buparlisib (BKM120)	Pan-PI3K inhibitor	Phase I	Novartis	NCT01396499
Dactolisib (BEZ235)	PI3K/mTOR inhibitor	Phase I	Novartis	NCT01756118
